# Ontogenetic Change in Male Expression of Testosterone-Responsive Genes Contributes to the Emergence of Sex-Biased Gene Expression in *Anolis sagrei*

**DOI:** 10.3389/fphys.2022.886973

**Published:** 2022-06-02

**Authors:** Matthew D. Hale, Christopher D. Robinson, Christian L. Cox, Robert M. Cox

**Affiliations:** ^1^ Department of Biology, University of Virginia, Charlottesville, VA, United States; ^2^ College of Arts, Sciences, and Education, Florida International University, Miami, FL, United States

**Keywords:** androgen, liver, ontogeny, RNAseq, sexual dimorphism, transcriptome

## Abstract

Sex differences in gene expression tend to increase with age across a variety of species, often coincident with the development of sexual dimorphism and maturational changes in hormone levels. However, because most transcriptome-wide characterizations of sexual divergence are framed as comparisons of sex-biased gene expression across ages, it can be difficult to determine the extent to which age-biased gene expression within each sex contributes to the emergence of sex-biased gene expression. Using RNAseq in the liver of the sexually dimorphic brown anole lizard (*Anolis sagrei*), we found that a pronounced increase in sex-biased gene expression with age was associated with a much greater degree of age-biased gene expression in males than in females. This pattern suggests that developmental changes in males, such as maturational increases in circulating testosterone, contribute disproportionately to the ontogenetic emergence of sex-biased gene expression. To test this hypothesis, we used four different experimental contrasts to independently characterize sets of genes whose expression differed as a function of castration and/or treatment with exogenous testosterone. We found that genes that were significantly male-biased in expression or upregulated as males matured tended to be upregulated by testosterone, whereas genes that were female-biased or downregulated as males matured tended to be downregulated by testosterone. Moreover, the first two principal components describing multivariate gene expression indicated that exogenous testosterone reversed many of the feminizing effects of castration on the liver transcriptome of maturing males. Collectively, our results suggest that developmental changes that occur in males contribute disproportionately to the emergence of sex-biased gene expression in the *Anolis* liver, and that many of these changes are orchestrated by androgens such as testosterone.

## Introduction

The evolution of sexual dimorphism presents a mechanistic challenge because the genomes producing dimorphic traits are overwhelmingly shared between males and females. Therefore, phenotypic sexual dimorphism usually requires sex differences in the expression of shared genes that underlie dimorphic traits (Ellegren and Parsch, 2007; Grath and Parsch, 2016). Both the number of genes that differ significantly in expression between the sexes and the magnitude of their sex-biased expression typically increase as ontogeny progresses, often coinciding with reproductive maturation and the emergence of phenotypic sexual dimorphism (Mank et al., 2010; Magnusson et al., 2011; Zhao et al., 2011; Ingleby, Flis and Morrow, 2015; Cox et al., 2017; Hale et al., 2018). However, most previous work has adopted a cross-sectional approach wherein the number of sex-biased genes or the magnitude of sex-biased expression is quantified at various ages, then compared across ages (e.g., Mank et al., 2010; Magnusson et al., 2011; Conforto and Waxman, 2012; Lowe et al., 2015; Cox et al., 2017; Hale et al., 2018). By contrast, relatively few studies have explored sex differences in gene expression by separately characterizing ontogenetic changes in gene expression within each sex, then comparing patterns of age-biased expression between sexes (but see Conforto and Waxman, 2012; Hou et al., 2017). Consequently, we have a limited understanding of how sex-biased gene expression emerges from ontogenetic changes within each sex, and whether developmental changes within one sex tend to contribute disproportionately to the emergence of sex-biased expression (e.g., Conforto and Waxman, 2012). Therefore, our first aim in this study was to address this knowledge gap by simultaneously comparing both sex-biased gene expression between ages and age-biased gene expression between sexes to determine the extent to which developmental changes occurring within each sex contribute to the ontogenetic emergence of sex-biased gene expression.

In the brown anole lizard (*Anolis sagrei*), previous work has found that sex-biased gene expression in the liver increases sharply as juveniles mature and the sexes diverge in body size, skeletal morphology, behavior, and ornamentation (Cox et al., 2017). The ontogenetic period over which sex-biased gene expression emerges in brown anoles implicates maturational effects of gonadal steroid hormones (e.g., androgens and estrogens), consistent with a large body of evidence linking androgens and estrogens to the development of sexual dimorphism in a variety of secondary sexual characteristics and across diverse vertebrate taxa (for reviews, see Roberts, Buchanan and Evans, 2004; Fusani, 2008; Jennings and de Lecea, 2020; Rey, 2021). In line with this view, previous work in brown anoles has shown that the steroid hormone testosterone induces the development of male-typical phenotypes (e.g., increased growth and body size, elevated metabolic rate, reduced fat storage, elaboration of the dewlap as a signaling ornament) in both males and females (Cox et al., 2009a, 2015, 2017; Wittman et al., 2021). Exogenous testosterone also masculinizes the liver transcriptome of juvenile females, particularly for growth-regulatory genes with naturally sex-biased expression, such as growth hormone receptor (*GHR*), insulin-like growth factors (*IGF1*, *IGF2*), and their receptors and binding proteins (Cox et al., 2017). While these data strongly implicate testosterone as a regulator of sex-biased gene expression for hepatic pathways related to growth, the extent to which testosterone shapes the broader, transcriptome-wide patterns of sex-biased gene expression in the liver that naturally emerge over ontogeny remains largely unknown. Our second aim in this study was therefore to experimentally characterize the responsiveness of gene expression in the liver to testosterone and then determine the extent to which patterns of sex-biased and age-biased gene expression can be predicted from responsiveness of gene expression to testosterone.

To achieve our first aim, we conducted differential gene expression analyses on liver transcriptomes from juveniles and subadults of each sex to characterize 1) significantly sex-biased genes at each age, and 2) significantly age-biased genes in each sex. A previous analysis of these same transcriptomes revealed a dramatic increase in the number of sex-biased genes between juvenile and subadult stages (Cox et al., 2017). We hypothesized that this ontogenetic emergence of sex-biased gene expression could be predicted from patterns of age-biased gene expression within each sex. We also tested whether patterns of age-biased gene expression differ between the sexes by asking whether females and males differed in the number of age-biased genes, the magnitude of age-biased expression, and the identity of genes and functional pathways with age-biased expression. To achieve our second aim, we used a combination of experimental methods to independently characterize genes that were responsive to castration and testosterone replacement in subadult males and to exogenous testosterone in juveniles of both sexes. To directly assess the role of testosterone in shaping ontogenetic changes in the male transcriptome and, by extension, ontogenetic increases in sex-biased gene expression, we tested whether responsiveness to testosterone predicted patterns of age-and sex-biased expression, and *vice versa*. Collectively, our study provides an example of how tissue-wide patterns of gene expression can be decomposed into separate readouts of sex-biased, age-biased, and hormonally regulated gene expression, then examined by characterizing the overlap among these different regulatory signatures.

## Materials and Methods

### Study Species and Experimental Design

We conducted experiments on brown anole lizards (*Anolis sagrei*) that were bred in captivity from adults collected in 2012 from the island of Great Exuma (23°29′N, 75°45′W) in The Commonwealth of the Bahamas. Anoles were collected with approval from the Bahamas Engineering, Science and Technology (BEST) Commission and the Ministry of Agriculture and imported with permission from the US Fish and Wildlife Service. All research was approved by the University of Virginia Animal Care and Use Committee (protocol 3896). Descriptions of experimental design and animal husbandry are provided in previous reports characterizing phenotypic responses to exogenous testosterone in juveniles (Cox et al., 2015) and effects of sex, age, and exogenous testosterone on hepatic gene expression (Cox et al., 2017). In this study, we reanalyze transcriptomes presented by Cox et al. (2017) alongside previously unpublished transcriptomes from subadult males that received castration and testosterone treatments. RNAseq reads are available *via* the NCBI Sequence Read Archive (PRJNA348684, PRJNA833864) and read counts from Cox et al. (2017) are available *via* Dryad (https://doi.org/10.5061/dryad.n95k3). All experiments were conducted on the same cohort and all transcriptomes were prepared and sequenced together, so published and previously unpublished transcriptomes are directly comparable with the caveat that experiments on the juvenile age class necessarily preceded those on the subadult age class by several months (see below).

At approximately 5 months of age, anoles that we refer to as “juveniles” were implanted with either a Silastic™ (Dow Corning, Midland, MI, United States) tubule containing 100 μg crystalline testosterone (T-1500, Sigma Aldrich Inc., St. Louis, MO, United States), or an empty implant as a control, then euthanized by decapitation at approximately 7 months of age (2 months post-treatment) to obtain RNA from liver tissue. Transcriptomes were prepared for a subset of juvenile females (*n* = 3) and juvenile males (*n* = 4) in the control groups, and for a subset of juvenile females (*n* = 3) and juvenile males (*n* = 4) that received testosterone implants. Additional details on animal husbandry, implant preparation, surgical procedures, circulating testosterone levels, and phenotypic effects of sex and testosterone treatment are provided by Cox et al. (2015). At approximately 12 months of age, anoles that we refer to as “subadults” were assigned to one of four treatment groups, then euthanized at approximately 14 months of age (2 months-post treatment) to obtain RNA from liver tissue. To facilitate direct comparison to juvenile controls, transcriptomes were prepared for intact subadult females (*n* = 4) and intact subadult males (*n* = 4) that received an empty implant. Transcriptomes were also prepared for two groups of subadult males that were castrated by surgical ablation of both testes, with one group then receiving an empty implant (castrated subadult males, *n* = 4) and the second group receiving an implant containing 300 μg crystalline testosterone (castrated subadult males + testosterone, *n* = 3). Livers were excised immediately following euthanasia, cut into 1-mm^3^ pieces, transferred into RNAlater (Qiagen, Valencia, CA, United States) on ice, held at 4°C for 24 h, then stored at −80°C until RNA extraction.

### Differential Expression Analysis

Liver transcriptomes were obtained as previously described (Cox et al., 2017). Briefly, cDNA libraries were generated from liver total RNA with NEB NextRNAseq kits (New England Biolabs; Ipswich, MA, United States), then sequenced twice, first on an Illumina MiSeq (300 bp single-end reads), then on a HiSeq 2500 (100 bp paired-end reads). Reads from both sequencing runs were combined for each individual and then mapped to the *Anolis carolinensis* transcript set (AnoCar2.0; Ensembl Release 75) using the BWA MEM algorithm (Li and Durbin, 2009). Unique alignments to *A. carolinensis* transcripts were counted *via* SAMtools (Li et al., 2009). Although read counting was done at the level of transcript (i.e., isoform), we refer to these transcripts as “genes” hereafter.

We conducted differential expression analysis using R (v3.6.1) (R Core Team, 2019) package edgeR (Robinson and Smyth, 2007; Robinson, McCarthy and Smyth, 2009; McCarthy, Chen and Smyth, 2012; Chen, Lun and Smyth, 2016). Prior to analysis, we filtered libraries to remove low-expression transcripts using edgeR function “FilterByExpr,” which retained 13,464 transcripts for analysis. Filtered read counts were then subjected to trimmed mean of *M*-values (TMM) normalization and fit to a negative binomial model in edgeR *via* function “glmQLFit” (robust = TRUE), followed by hypothesis testing using quasi-likelihood *F*-tests in planned linear contrasts (function “glmQLFTest”). We considered any transcript with a Benjamini and Hochberg, (1995) false discovery rate-adjusted *p* < 0.05 (hereafter, FDR < 0.05) to be a differentially expressed gene (DEG).

We characterized age-biased gene expression within each sex by contrasting 1) juvenile females vs. subadult females, and 2) juvenile males vs. subadult males. Sex-biased gene expression has previously been described for this dataset (Cox et al., 2017), but to facilitate direct comparison to our new age-biased measures, we used the same parameters and filtering steps described above to re-characterize sex-biased gene expression at each age by contrasting 3) juvenile females vs. juvenile males, and 4) subadult females vs. subadult males. We independently characterized effects of testosterone on gene expression by contrasting 5) juvenile females with empty implants vs. juvenile females with testosterone implants; 6) juvenile males with empty implants vs. juvenile males with testosterone implants, 7) castrated subadult males with empty implants vs. intact subadult males with empty implants, and 8) castrated subadult males with empty implants vs. castrated subadult males with testosterone implants. Because relatively few DEGs were identified as testosterone-responsive when using a strict FDR cutoff, we broadened our classification to include all genes with an uncorrected *p* < 0.01, which provided us with a sufficiently large sample of DEGs from each experimental contrast to subsequently test whether responsiveness to testosterone predicted patterns of sex- and age-biased gene expression. To visualize differences in gene expression for each of the eight pairwise contrasts described above, we created volcano plots with the unadjusted log_10_ *p* value for each gene plotted against the log_2_ fold change (FC) in expression between the two groups. Summary statistics for each gene in each experimental contrast (e.g., log_2_ FC, *F* statistics, and *p* values) are available as Supplementary Material. Gene symbols (names) were obtained by converting original transcript identifiers (AnoCar2.0; Ensembl Release 75) to current (AnoCar2.0v2; Ensembl Release 106) identifiers *via* biomaRt. HGNC gene symbols were included where available for any transcript with a stable identifier across both releases.

### Linking Sex Bias, Age Bias, and Regulation by Testosterone

First, to determine whether genes with age-biased expression in each sex exhibited predicted patterns of sex-biased expression in subadults, we tested whether DEGs with either subadult-biased expression in males or juvenile-biased expression in females had significantly positive (i.e., male-biased) log_2_ (FC) values for sex bias, and whether DEGs with either subadult-biased expression in females or juvenile-biased expression in males had significantly negative (i.e., female-biased) log_2_ (FC) values for sex bias. To do this, we tested whether these age-biased DEGs had mean and median log_2_ (FC) values for sex bias in subadults that differed from a null hypothesis of log_2_ (FC) = 0 (i.e., no consistent sex bias) using Welch’s one-sample *t*-tests (mean) or Wilcoxon signed-rank tests (median). We also made the reciprocal comparisons by testing whether sex-biased DEGs exhibited predicted patterns of age-biased expression in each sex. These various comparisons are non-independent in the sense that the subadult males and females used to characterize sex-biased expression are the same transcriptomes used to characterize age-biased expression within each sex. Therefore, these analyses should be interpreted as descriptions of the extent to which sex bias and age bias overlap, not as formal hypothesis tests.

Second, to test whether changes in gene expression with age tend to be similar in males and females (i.e., whether genes that are upregulated in subadult males are also upregulated in subadult females), we used Welch’s *t*-tests and Wilcoxon signed-rank tests (see above) to test whether subadult-biased DEGs from males had significantly positive (i.e., subadult-biased) mean and median log_2_ (FC) values in females, and whether juvenile-biased DEGs from males had significantly negative (i.e., juvenile-biased) mean and median log_2_ (FC) values in females. We also made the reciprocal comparisons by testing whether age-biased DEGs from females had significantly positive or negative mean and median log_2_ (FC) values in males. Age-biased DEGs were identified in one sex and then tested against log_2_ (FC) values derived from entirely different transcriptomes from the other sex, so there is no problem of non-independence for statistical comparisons between sexes.

Third, to test whether sex- and age-biased genes are regulated by testosterone, we used Welch’s *t*-tests and Wilcoxon signed-rank tests (see above) to test whether DEGs inferred to be upregulated by testosterone in any of our four experimental contrasts had significantly positive mean and median log_2_ (FC) values for sex bias in subadults (i.e., male-biased expression) and for age bias in males (i.e., subadult-biased expression). Likewise, we tested whether DEGs inferred to be downregulated by testosterone had significantly negative mean and median log_2_ (FC) values for sex bias in subadults (i.e., female-biased expression) and for age bias in males (i.e., juvenile-biased expression). We conducted reciprocal comparisons by testing whether male-biased DEGs in subadults and subadult-biased DEGs in males had significantly positive mean and median log_2_ (FC) values in experimental comparisons (i.e., upregulated by testosterone), and whether female-biased DEGs in subadults and juvenile-biased DEGs in males had significantly negative mean and median log_2_ (FC) values in experimental comparisons (i.e., downregulated by testosterone). Of the 16 different statistical tests generated by either of these approaches, 6 are based on non-independent classifications of expression patterns (i.e., both classifications involve either juvenile males with empty implants or subadult males with empty implants as a point of reference), but the other 10 tests involve independent datasets. Due to the large number of independent comparisons, we used Bonferroni corrections to adjust significance thresholds within each of the three main comparisons (and their three reciprocal approaches) described above.

### Log Fold Change Distribution

To supplement our use of differentially expressed genes (DEGs) as a metric of sex and age bias, we also characterized quantitative differences in the distributions of log_2_ (FC) values. To test whether the magnitude of sex-biased gene expression differed between juveniles and subadults, we tested whether the median absolute value of log_2_ (fold change between sexes) differed between juveniles and subadults using a Wilcoxon rank sum test. We also compared the variance in log_2_ (fold change between sexes) between juveniles and subadults using Bartlett’s test for equal variances (*α* = 0.05). Similarly, we used Wilcoxon and Bartlett’s tests to assess whether the sexes differed in the median absolute value and variance in log_2_ (fold change between juveniles and subadults).

### Gene Ontology Enrichment

To predict biological functions attributable to DEGs, we used gene ontology (GO) term enrichment analyses (GO: biological process). We conducted these analyses using g:Profiler against a liver-specific background (all 13,464 genes passing filtering) over all known genes with a g:SCS multiple-testing correction approach for ontology analysis (Reimand et al., 2016). Prior to analyses, *A. sagrei* liver transcripts (i.e., ENSACAT) were converted to human orthologs (i.e., ENSG) *via* Ensembl BioMart (Howe et al., 2021). For each of the eight contrasts described above, we tested for pathway enrichment separately for DEGs with negative vs. positive log_2_ (FC) scores (i.e., separately testing for enrichment with female- or male-biased genes, juvenile- or subadult-biased genes, and genes that were down or upregulated by testosterone).

### Principal Components Analysis

We used principal components analysis (PCA) to describe multivariate patterns of gene expression and test for transcriptome-wide effects of age, sex, and testosterone treatment. Prior to PCA, all read counts were normalized using a median of ratios (MR) method and transformed with a regularized log (“rlog,” blind = FALSE) transformation in R package DESeq2 (Love, Huber and Anders, 2014). We conducted PCA using R function “prcomp” (scale = TRUE). First, to characterize effects of sex and age independently of testosterone manipulation, we conducted PCA with filtered read counts from only those individuals with intact gonads and empty implants (i.e., juvenile females, juvenile males, subadult females, and subadult males). Second, to characterize effects of testosterone treatment in juveniles, we conducted a similar PCA using only the four juvenile groups (i.e., juveniles of each sex with empty implants, juveniles of each sex with testosterone implants). Third, to characterize effects of castration and testosterone replacement in subadults, we conducted PCA using only the four subadult groups (i.e., intact subadult females and males with empty implants, castrated subadult males with either empty or testosterone implants). Finally, we conducted a holistic PCA analysis including all eight groups. For each analysis, we used the first and second principal components (PC1 and PC2) to describe the major axes of variation in gene expression across groups. For the first analysis, we tested for effects of sex, age, and the sex*age interaction using 2-way ANOVA conducted separately on PC1 and PC2 values. For the second analysis, we tested for effects of sex, testosterone treatment, and the sex*treatment interaction using 2-way ANOVA conducted separately on PC1 and PC2 values. For the final two analyses, treatments were not balanced across sex and age, so we used 1-way ANOVA with Tukey’s post-hoc tests to characterize differences in PC1 and PC2 across the various sex, age, and treatment groups.

### Targeted Analysis of Growth Pathways

To describe patterns of sex bias, age bias, and responsiveness to testosterone in growth-regulatory gene networks, we characterized log_2_ (FC) values for *n* = 12 genes in the growth hormone/insulin-like growth factor (GH/IGF) pathway and for *n* = 23 genes in the mechanistic target of rapamycin (mTOR) pathway, using the same pathway members as Cox et al. (2017). Both pathways have previously been shown to exhibit sex-biased expression in the anole liver (Cox et al., 2017). We plotted log_2_(FC) values for these genes separately for each our 8 experimental contrasts (2 for sex bias, 2 for age bias, 4 for responsiveness to testosterone), then highlighted genes that were significant DEGs in at least one of the 8 statistical contrasts to compare their patterns of expression across other contrasts.

## Results

### Sex- and Age-Biased Gene Expression

**FIGURE 1:**
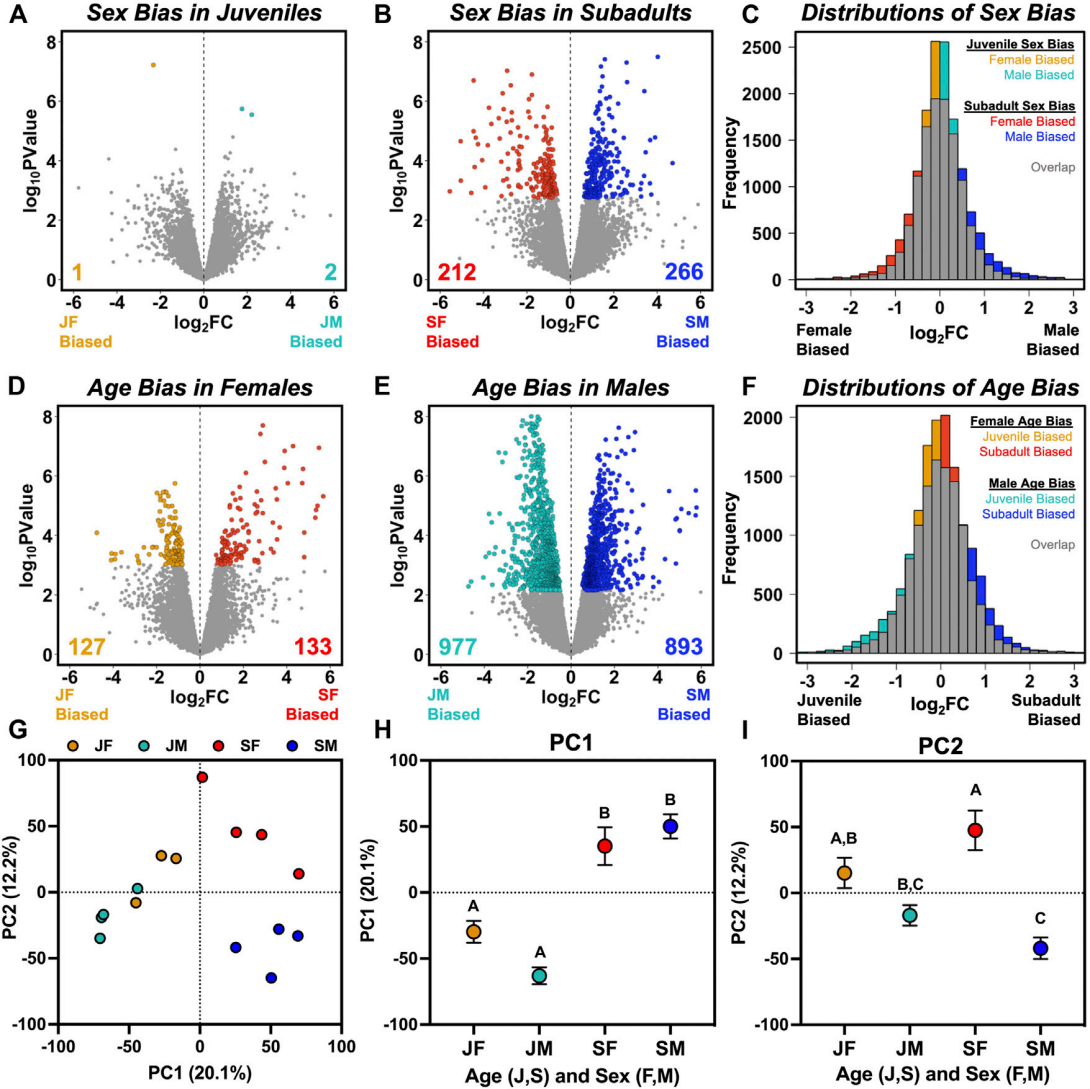
Visualization of sex-and age-biased gene expression based on comparisons of juvenile females (JF), juvenile males (JM), subadult females (SF), and subadult males (SM). **(A,B)** Volcano plots with colored symbols indicating significantly (FDR < 0.05) sex-biased genes in **(A)** juveniles, and **(B)** subadults, with unbiased genes in gray. **(C)** Frequency distributions of log_2_ fold change (FC) values from **(A,B)**, with gray indicating overlap between juvenile and subadult distributions and colors indicating an excess of low sex bias in juveniles and high sex bias in subadults. **(D,E)** Volcano plots with colored symbols indicating significantly (FDR < 0.05) age-biased genes in **(D)** females, and **(E)** males, with unbiased genes in gray. **(F)** Frequency distributions of log_2_ (FC) values from **(D,E)**, with gray indicating overlap between female and male distributions and colors indicating an excess of low age bias in females and high age bias in males. **(G)** Separation of individuals along the first two principal components describing variation in transcriptome-wide expression, alongside the mean (±SE) values for **(H)** PC1, and **(I)** PC2 for each group. Letters above each symbol indicate post-hoc separation based on analysis of variance.

We observed a relatively low degree of sex-biased gene expression between juvenile females and juvenile males, with only three significantly (FDR < 0.05) differentially expressed genes (DEG; Figure 1A). One of these DEGs with male-biased expression is *FADS2*, which encodes fatty acid desaturase 2, while the other two are predicted to encode proteins but are not annotated in the *A. carolinensis* genome, although one (ENSACAT00000022830) is partially orthologous to *COG2*, a subunit member of the oligermic golgi complex, in other reptile species.

The overall lack of sex-biased transcription in juveniles contrasts sharply with subadults, in which we found 478 significant DEGs (Figure 1B). When considering quantitative sex bias in gene expression rather than qualitative assignment of DEGs based on statistical thresholding, subadults exhibited a higher median absolute value of log_2_ (FC) between sexes than juveniles (subadult median = 0.373; juvenile median = 0.280; Wilcoxon *p* < 0.0001), and a significantly greater variance in log_2_ (FC) between sexes than juveniles (subadult *σ* ^2^ = 0.551; juvenile *σ* ^2^ = 0.320; Bartlett’s *K* ^2^ = 989.05; *p* < 0.0001; Figure 1C). GO enrichment analysis was not conducted for juveniles due to the low number of sex-biased genes. In subadults, biological pathways enriched with male-biased genes included regulation of coagulation, hemostasis, humoral immunity, and peptidase activity, whereas we did not find any biological pathways enriched with female-biased genes (Supplementary Figure S1A).

When characterizing age bias within a sex (rather than sex bias within an age), we found 260 age-biased DEGs in females (Figure 1D) and 1870 age-biased DEGs in males (Figure 1E), an approximately 7-fold difference between sexes. Although this difference could be partly due to unequal sample sizes (*n* = 3 juvenile females, *n* = 4 for other groups), we still found >1,380 significantly age-biased DEGs in males in each of four additional analyses in which we iteratively excluded one of the four juvenile male libraries, such that males always exceeded females by more than 5-fold in total number of age-biased genes (Supplementary Table S1). Males also exhibited a higher median absolute value of log_2_ (FC) between ages than females (male median = 0.455; female median = 0.319; Wilcoxon *p* < 0.0001), and a significantly greater variance in log_2_ (FC) between ages than females (male *σ* ^2^ = 0.652; female *σ* ^2^ = 0.502; Bartlett’s *K* ^2^ = 230.13; *p* < 0.0001; Figure 1F). In males, juvenile-biased DEGs were enriched in pathways related to development and morphogenesis, whereas subadult-biased DEGs were enriched in pathways related to metabolism and translation (Supplementary Figure S1B). In females, we could not detect enrichment of juvenile-biased DEGs in any biological pathway, but subadult-biased DEGs were enriched in pathways related to ribosome biogenesis, which were also enriched for subadult-biased DEGs in males (Supplementary Figure S1B).

Principal components analysis indicated that age contributed relatively more to overall variation in transcriptome-wide expression than did sex (Figure 1G). PC1 accounted for 20% of variation in expression and 2-way ANOVA for PC1 values identified a large effect of age (*F*_1,11_ = 73.54; *p* < 0.0001), no effect of sex (*F*_1,11_ = 0.78 *p* = 0.396), and a weak interaction between age and sex (*F*_1,11_ = 5.39; *p* = 0.040; Figure 1H). PC2 accounted for 12% of variation in gene expression and separated samples primarily by sex (*F*_1,11_ = 29.83; *p* = 0.0002) and its interaction with age (*F*_1,11_ = 6.63; *p* = 0.026), but not by age alone (*F*_1,11_ = 0.11; *p* = 0.74; Figure 1I). Tukey’s post-hoc tests showed that juveniles and subadults were statistically distinct for PC1, but that the sexes did not differ for PC1 at either age point (Figure 1H), and that subadult males and females differed for PC2, but juvenile males and females did not (Figure 1I).

### Linking Sex- and Age-Biased Gene Expression

**FIGURE 2:**
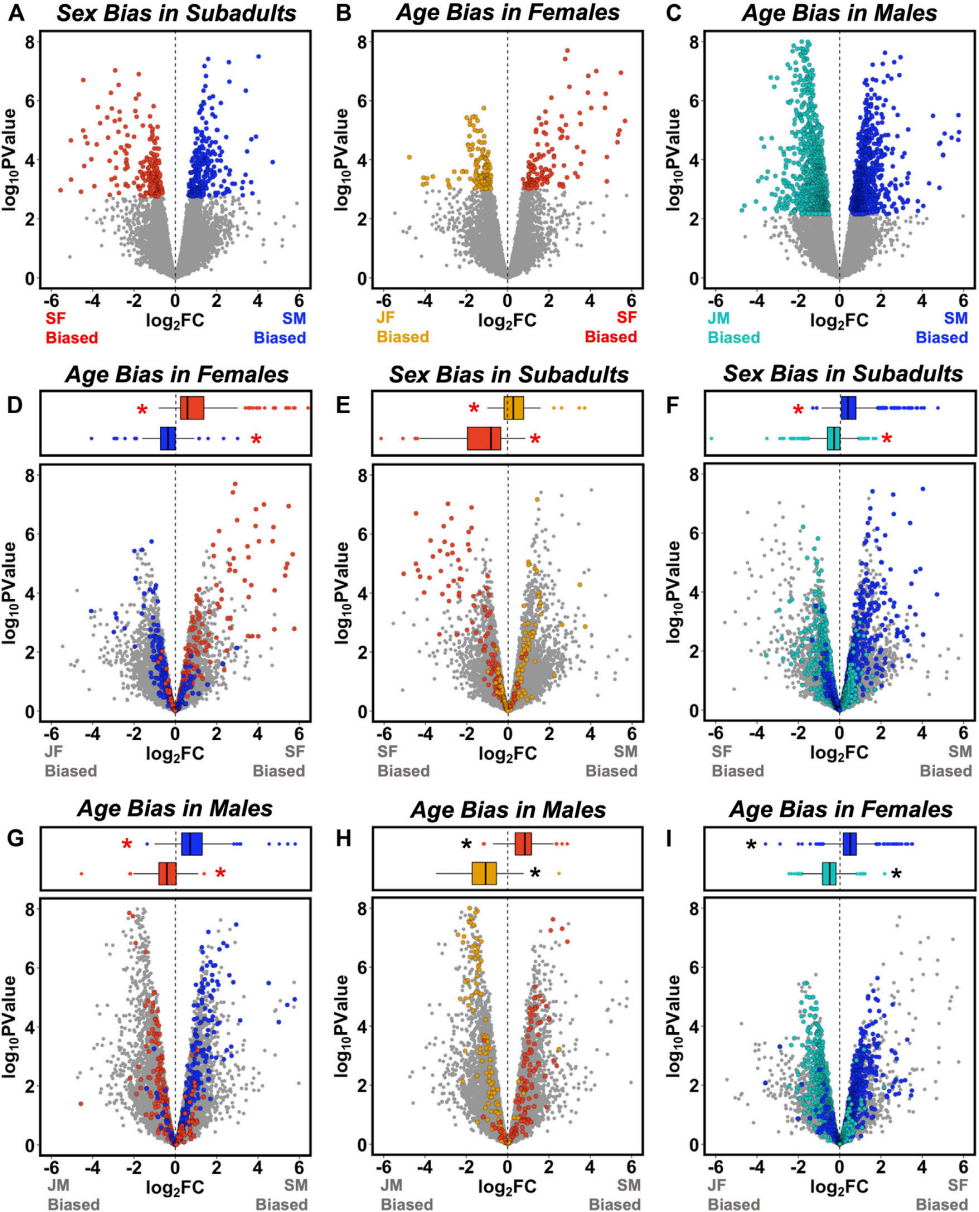
Overlap between sex-and age-biased gene expression. Volcano plots in the top row illustrate patterns of **(A)** sex-biased expression in subadults, **(B)** age-biased expression in females, and **(C)** age-biased expression in males, with differentially expressed genes (DEGs, FDR <0.05) shown in color and other genes in gray (as in Figure 1). In the left column, sex-biased DEGs from **(A)** are mapped onto plots describing age-biased expression in **(D)** females, and **(G)** males. In the middle column, female age-biased DEGs from **(B)** are mapped onto plots describing **(E)** sex-biased expression, and **(H)** age-biased expression in males. In the right column, male age-biased DEGs from **(C)** are mapped onto plots describing **(F)** sex-biased expression, and **(I)** age-biased expression in females. Box-and-whisker plots above each panel report the median (line), upper 75% and lower 25% (box), and upper 95% and lower 5% (whiskers) for the log_2_ fold change (FC) values of each category of DEG [from **(A–C)**] when mapped onto the corresponding volcano plots. Asterisks indicate mean log_2_ FC values significantly different from zero following Bonferroni correction for multiple comparisons (adjusted *p* < 0.0042). Red asterisks indicate tests for which the classification of DEGs is not statistically independent of the log_2_ (FC) values on the volcano plot because the same group is included in each comparison.

When genes that were significantly sex-biased in subadults (Figure 2A) were tested for age-biased expression in each sex (Supplementary Table S2), we found that male-biased DEGs were significantly juvenile-biased in females (Figure 2D) and subadult-biased in males (Figure 2G), whereas female-biased DEGs were significantly subadult-biased in females (Figure 2D) and juvenile-biased in males (Figure 2G). The reciprocal approach yielded similar results (Supplementary Table S2). When genes that were significantly age-biased in females (Figure 2B) were tested for sex-biased expression in subadults, juvenile-biased DEGs were significantly male-biased (Figure 2E), whereas subadult-biased DEGs were significantly female-biased (Figure 2E). Likewise, when genes that were significantly age-biased in males (Figure 2C) were tested for sex-biased expression in subadults, juvenile-biased DEGs were significantly female-biased (Figure 2F), whereas subadult-biased DEGs were significantly male-biased (Figure 2F). Approximately half (233 of 478; 49%) of the significantly sex-biased DEGs in subadults were also identified as significantly age-biased DEGs in either males or females (Supplementary Figure S2A). Although these associations are expected in part because the same subadult transcriptomes that we used to identify sex-biased DEGs were also used to characterize age-biased expression in each sex, these statistical associations nonetheless confirm an important assumption of our approach (i.e., that patterns of age bias within each sex capture features of the development of sex-biased gene expression).

However, associations between age bias and sex bias were not perfect. For example, some genes that were significantly upregulated with age in one sex were nonetheless sex-biased toward greater expression in subadults of the opposite sex (Figures 2E,F). Consistent with this observation, we found that patterns of age-biased expression were broadly similar in each sex. When genes that were significantly age-biased in females (Figure 2B) were tested for age-biased expression in males (Supplementary Table S2), juvenile-biased DEGs were significantly juvenile-biased (Figure 2H) and subadult-biased DEGs were significantly subadult-biased (Figure 2H). Likewise, when genes that were significantly age-biased in males (Figure 2C) were tested for age-biased expression in females (Supplementary Table S2), juvenile-biased DEGs were significantly juvenile-biased (Figure 2I) and subadult-biased genes were significantly subadult-biased (Figure 2I). Relatively few genes exhibited strongly opposing patterns of age-biased expression across sexes (Figures 2H,I).

Five genes were significantly age-biased in both sexes and also significantly sex-biased in subadults (Supplementary Figure S2A), including heat-shock factor 3 (*HSF-3*), transmembrane protein with EGF-like and two follistatin-like domains 1 (*TMEFF1*), nucleolar protein 56 (*NOP56*); ALG1 chitobiosyldiphosphodolichol beta-mannosyltransferase; (*ALG1*), and alpha-1-antitrypsin (*SERPINA1*). Only two of these genes (*HSF-3* and *TMEFF1*) exhibited opposing patterns of age bias in each sex, and their relationship to phenotypic sexual dimorphism is not clear, although *TMEFF1* has diverse functions including regulation of development, metabolism, and endocrine function (Labeur et al., 2015; Masood et al., 2020). *HSF3* is an inducible transcription factor that participates in acute stress and heat-shock response (Åkerfelt, Morimoto and Sistonen, 2010).

### Effects of Testosterone on Gene Expression

**FIGURE 3:**
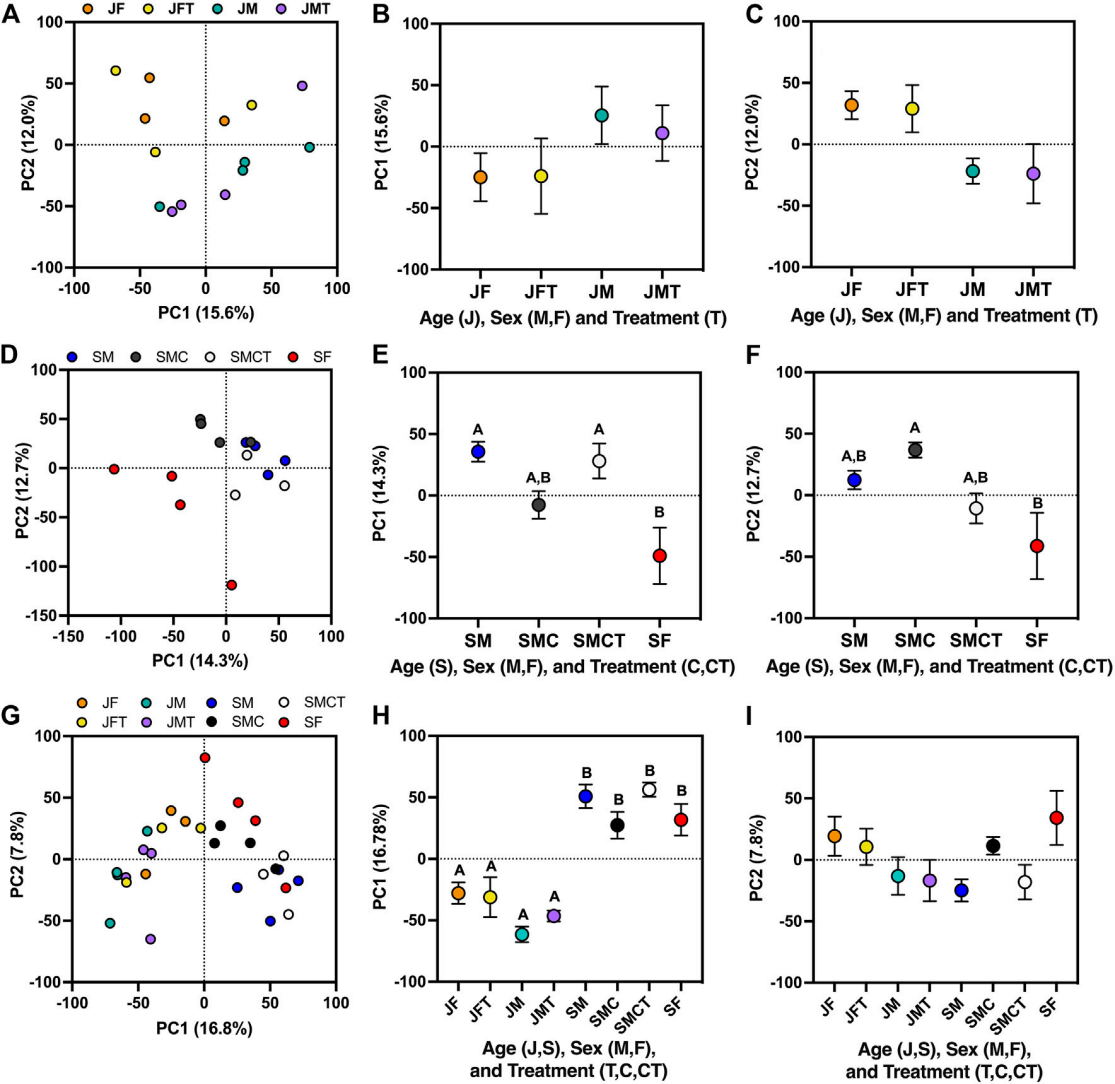
Principle components analyses illustrating effects of testosterone on multivariate patterns of gene expression. **(A)** Separation of samples from juvenile females and males with empty implants (JF, JM) or testosterone implants (JFT, JMT) along the first two principal components, alongside mean (±SE) values for **(B)** PC1, and **(C)** PC2 in each group. Two-way ANOVA revealed a significant effect of sex on PC2 values (not shown). **(D)** Separation of samples from subadult females and males with empty implants (SF, SM) and castrated subadult males with empty implants or testosterone implants (SMC, SMCT) along PC1 and PC2, alongside mean (±SE) values for **(E)** PC1, and **(F)** PC2 in each group. Letters above each symbol indicate post-hoc separation based on one-way ANOVA. **(G)** Separation of samples from each of the eight juvenile and subadult groups described above along PC1 and PC2, alongside mean (±SE) values for **(H)** PC1, and **(I)** PC2 in each group.

Effects of testosterone on gene expression in juveniles were relatively weak when assessed *via* PCA (Figures 3A–C). PC1 and PC2 collectively explained 27% of the variance in gene expression and weakly separated samples by sex (PC1: *F*_1,10_ = 3.02, *p* = 0.11; PC2: *F*_1,10_ = 8.65, *p* = 0.015), with no significant effects of treatment (PC1: *F*_1,10_ = 0.08, *p* = 0.79; PC2: *F*_1,10_ = 0.02, *p* = 0.89) or the sex*treatment interaction (PC1: *F*_1,10_ = 0.10, *p* = 0.76; PC2: *F*_1,10_ < 0.01, *p* = 0.98). In a separate PCA involving only subadults, PC1 and PC2 also collectively explained 27% of the variance in gene expression (Figures 3D,E). On PC1, intact subadult males and castrated subadult males with testosterone implants were similar to one another (Tukey’s *p* = 0.986) and also statistically distinct from subadult females (*p* = 0.009 and *p* = 0.027, respectively), whereas castrated subadult males without testosterone implants were statistically indistinguishable from subadult females (*p* = 0.259; Figure 3E). On PC2, intact subadult males were again similar to castrated subadult males with testosterone implants (*p* = 0.772), but these two groups were intermediate between castrated males and intact females, which were statistically distinct (*p* = 0.021; Figure 3F). When all eight experimental groups were analyzed together in a single PCA, PC1 explained 17% of the variance in gene expression and separated samples primarily by age (*p* < 0.05 for all post hoc pairwise comparisons across age), with subadult females and castrated subadult males more similar to juveniles than were intact subadult males or castrated subadult males with testosterone implants (Figure 3H). PC2 explained 8% of the variance in expression and separated samples primarily by sex, although no post hoc comparisons were significant for PC2. However, castrated males without testosterone replacement exhibited positive PC2 values like those of females, whereas all other male groups exhibited negative values (Figure 3I).

**FIGURE 4:**
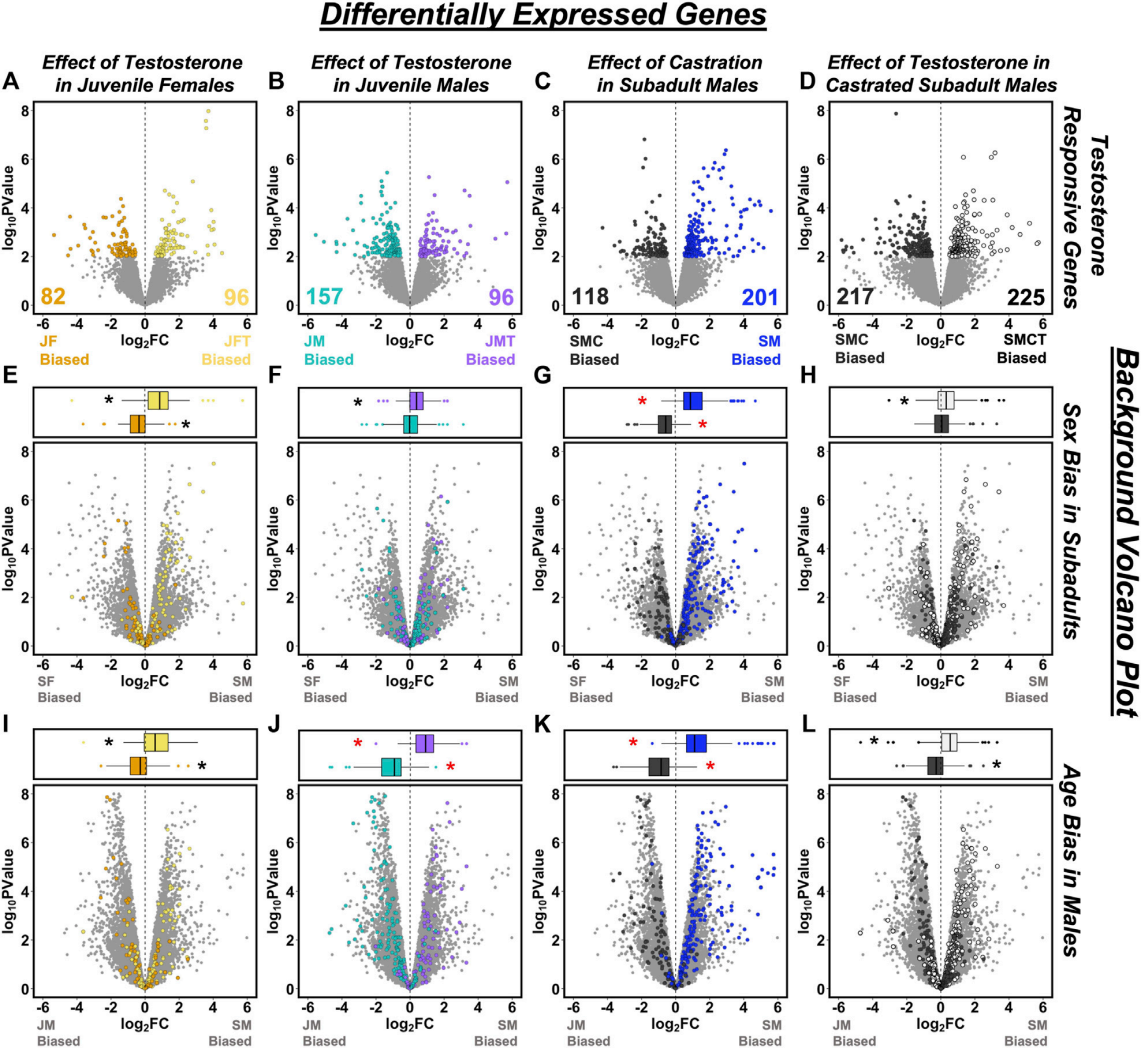
Overlap between testosterone-responsive genes and patterns of sex- or age-biased expression. Volcano plots in the top row illustrate effects of testosterone on gene expression based on comparisons of **(A)** juvenile females with empty vs. testosterone implants (JF vs. JFT), **(B)** juvenile males with empty vs. testosterone implants (JM vs. JMT), **(C)** castrated vs. intact subadult males (SMC vs. SM), and **(D)** castrated subadult males with empty vs. testosterone implants (SMC vs. SMCT). Differentially expressed genes (DEGs, *p* < 0.01) are plotted in color and other genes are in gray. In the bottom two rows, testosterone-responsive DEGs from **(A–D)** are mapped onto plots describing **(E–H)** sex-biased expression in subadults, and **(I–L)** age-biased expression in males. Box-and-whisker plots above each panel report the median (line), upper 75% and lower 25% (box), and upper 95% and lower 5% (whiskers) for the log_2_ fold change (FC) values of each category of DEG when mapped onto the corresponding volcano plot. Asterisks indicate mean log_2_ FC values significantly different from zero following Bonferroni correction for multiple comparisons (adjusted *p* < 0.0031). Red asterisks indicate tests for which the classification of DEGs is not statistically independent of the log_2_ (FC) values on the volcano plot because the same group is included in each comparison.

When using a strict FDR cutoff to identify testosterone-responsive genes, we found only 8 DEGs between juvenile females with empty vs. testosterone implants, 14 DEGs between juvenile males with empty vs. testosterone implants, 34 DEGs between castrated vs. intact subadult males, and 9 DEGs between castrated subadult males with empty vs. testosterone implants. Lowering the significance threshold to an uncorrected *p <* 0.01 increased the number of DEGs in each comparison to 178 (Figure 4A), 253 (Figure 4B), 319 (Figure 4C), and 442 (Figure 4D), respectively. Although the genes that we identified as DEGs did not overlap substantially across the four different experimental contrasts used to infer responsiveness to testosterone (Supplementary Figure S2B), the DEGs identified by any given contrast consistently displayed the same general patterns of responsiveness to testosterone across each of the other three contrasts (Supplementary Figure S3; Supplementary Table S3).

Eight testosterone-responsive genes were consistently identified as significant (*p* < 0.01) DEGs across all four experimental contrasts. Two of these DEGs are transcripts expressed from the same gene, sex hormone-binding globulin (*SHBG*), both of which were consistently suppressed by testosterone. Other DEGs that consistently responded to testosterone include fibulin 1 (*FBLN1*), diphosphoinositol pentakisphosphate kinase 2 (*PPIP5K2*); NHL repeat containing 4 (*NHLRC4*), reticulophagy regulator family member 3 (*RETREG3*); tubulin beta-7 chain-like (*LOC100558555*), and an uncharacterized protein with predicted oxidoreductase activity (ENSACAT00000017273). Of these, only *LOC100558555* was suppressed by testosterone.

Relatively few functional pathways were enriched for the DEGs identified as responsive to testosterone. One pathway related to phosphatidylinositol biosynthesis (GO:0046854) was enriched for genes upregulated in response to testosterone in juvenile males, and the KEGG “ribosome” pathway (KEGG:03010, which is included within some of the ribosome-related pathways that were enriched for subadult-biased genes in males) was enriched for DEGs that were expressed more highly in intact subadult males relative to castrated subadult males.

### Linking Sex- and Age-Biased Gene Expression to Testosterone

When genes that were differentially expressed (*p* < 0.01) in the four experimental contrasts (Figures 4A–D) were tested for sex-biased gene expression in subadults, we found that DEGs inferred to be upregulated by testosterone were significantly male-biased in each case (Figures 4E–H; Supplementary Table S4). We also observed the predicted pattern of significantly female-biased expression for DEGs that were downregulated by exogenous testosterone in juvenile females or upregulated by castration in subadult males (Figures 4E,G; Supplementary Table S4), but not for DEGs that were downregulated by exogenous testosterone in either juvenile males or castrated subadult males (Figures 4F,H; Supplementary Table S4). When these same sets of testosterone-responsive genes were tested for age-biased expression in males, we found that DEGs inferred to be upregulated by testosterone were significantly subadult-biased in all cases (Figures 4I–L; Supplementary Table S4), whereas DEGs inferred to be downregulated by testosterone were significantly juvenile-biased in all cases (Figures 4I–L; Supplementary Table S4). Therefore, responsiveness to testosterone predicted patterns of sex-biased expression in 6 of 8 tests and it predicted patterns of age-biased expression in males in 8 of 8 tests (Supplementary Table S4).

Reciprocal analyses generated similar results (Supplementary Table S5). When significantly (FDR < 0.05) age-biased genes in males were tested for responsiveness to testosterone, subadult-biased DEGs were significantly upregulated by testosterone in 3 of 4 comparisons (Supplementary Figures S4A–D), whereas juvenile-biased DEGs were significantly downregulated by testosterone in 4 of 4 comparisons (Supplementary Figures S4A–D). When significantly (FDR < 0.05) sex-biased genes were tested for their responsiveness to testosterone, male-biased DEGs were significantly upregulated by testosterone in 4 of 4 cases (Supplementary Figure S4E–H), whereas female-biased DEGs were significantly downregulated by testosterone in 2 of 4 cases (Supplementary Figure S4E–H).

### Targeted Analysis of Growth Pathways

**FIGURE 5:**
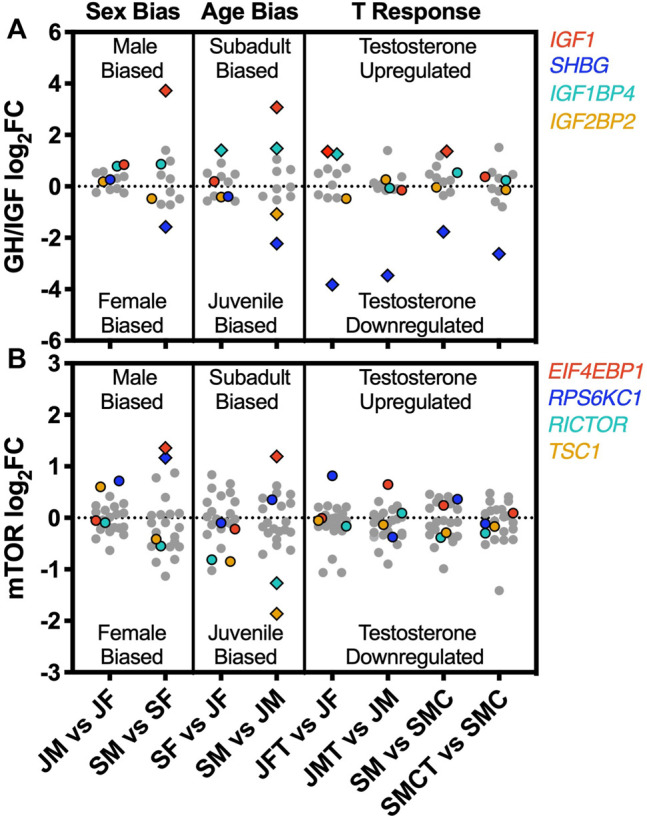
Sex bias, age bias, and responsiveness to testosterone for genes in two growth-regulatory pathways. Log_2_ (FC) values for genes in the **(A)** growth hormone/insulin-like growth factor (GH/IGF) and **(B)** mechanistic target of rapamycin (mTOR) signaling pathways, shown separately for each of eight experimental comparisons illustrating sex bias (juvenile males vs. juvenile females, JM vs. JF; subadult males vs. subadult females, SM vs. SF), age bias (subadult females vs. juvenile females, SF vs. JF; subadult males vs. juvenile males, SM vs. JM), and responsiveness to testosterone (juvenile females with and without testosterone: JFT vs. JF; juvenile males with and without testosterone, JMT vs. JM; intact subadult males vs. castrated subadult males, SM vs. SMC; castrated subadult males with and without testosterone, SMCT vs. SMC). Colored symbols represent genes detected as a significant DEG in at least one of the 8 contrasts. Diamond symbols represent genes detected as DEGs within a particular contrast. Grey symbols were not significantly differentially expressed in any contrast.

Four of the 12 genes in the GH/IGF pathway were identified as DEGs in at least one of our 8 experimental contrasts, including insulin-like growth factor 1 (*IGF1*), insulin-like growth factor binding protein 4 (*IGF1BP4*), insulin-like growth factor 2 mRNA binding protein 2 (*IGF2BP2*), and sex hormone-binding globulin (*SHBG*). All of these genes were significantly age-biased in males, whereas only one (*IGF1BP4*) was also age-biased in females (Figure 5A). Testosterone upregulated expression of *IGF1* and *IGF1BP4*, although the strength of this effect varied across different contrasts (Figure 5A), and testosterone consistently suppressed the expression of *SHBG* (Figure 5A). Four of 23 genes in the mTOR pathway were identified as DEGs in at least one contrast, including eukaryotic translation initiation factor 4E binding protein (*EIF4EBP1*), ribosomal protein s6 kinase (*RPS6KC1*), RPTOR independent companion of MTOR, complex 2 (*RICTOR*), and tuberous sclerosis 1 (*TSC1*). Three of these genes were age-biased in males (*EIF4EBP1*, *RICTOR*, and *TSC1*), two were sex-biased in subadults (*EIF4EBP1* and *RPS6KC1*), and none were significantly responsive to testosterone (Figure 5B).

## Discussion

We found that the sharp ontogenetic increase in sex-biased gene expression in the brown anole liver that was previously reported by Cox et al. (2017) is associated with a substantially greater developmental change in the hepatic transcriptome of males, relative to that of females. Similar results have been reported for the mouse liver, where sex-biased genes in adults are more likely to exhibit age-biased expression in males than in females (Conforto and Waxman, 2012). In brown anoles, this pronounced sex difference in age-biased gene expression is evident in both the number of significantly age-biased genes and in the transcriptome-wide distribution of log_2_ (FC) values for age bias. Despite these clear quantitative sex differences, the direction of age bias appears to be broadly similar between females and males (i.e., the same genes tend to be consistently up or downregulated with age in both sexes). Therefore, the emergence of sex-biased gene expression in the brown anole liver appears to be primarily attributable to quantitative sex differences in the degree of up or downregulation with age, rather than widespread sex differences in the direction of regulatory change with age. Sex-biased gene expression often emerges as animals develop towards sexual maturity (Mank et al., 2010; Magnusson et al., 2011; Zhao et al., 2011; Grath and Parsch, 2016; Hale et al., 2018). Yet, despite growing recognition that ontogenetic characterizations of sex-biased transcription are needed to fully understand the temporal dynamics of sexual dimorphism (Mank et al., 2010; Mank and Rideout, 2021), studies that decompose cross-sectional measures of sex bias at particular life stages into sex-specific developmental changes in transcription are rare (Conforto and Waxman, 2012; Hou et al., 2017). Our study calls attention to the general point that the sexes may often contribute asymmetrically to the ontogenetic emergence of sex-biased gene expression, while also raising the more specific hypothesis that developmental changes that occur in males are particularly important to this process in the brown anole liver.

In support of this specific hypothesis, we found that genes that are upregulated by testosterone are more likely to be both male-biased and upregulated with age in males, whereas genes that are downregulated by testosterone are more likely to be female-biased and downregulated with age in males. We observed this same general pattern when using a variety of independent experimental contrasts to characterize the responsiveness of gene expression to exogenous testosterone in juveniles of both sexes and following castration and testosterone replacement in subadult males. Our four experimental contrasts were broadly congruent in the overall patterns of responsiveness to testosterone that they revealed, although the majority of individual DEGs identified as responsive to testosterone were unique to a single experimental contrast. This could reflect error variance due to low sample sizes within treatment groups (*n* = 3 or 4), or biological differences in responsiveness to testosterone across different sexes and ages. Nonetheless, the fact that each contrast generated sets of up and downregulated genes that independently predicted patterns of sex-biased and age-biased expression provides broad overall support for a regulatory role of testosterone. Therefore, we conclude that maturational increases in testosterone likely structure ontogenetic changes in the liver transcriptome of males, thereby contributing to the development of sex-biased gene expression with age.

Several caveats are important to note. First, we found relatively few genes differentially expressed in response to exogenous testosterone or castration when applying a rigorous cutoff for false discovery. In part, this likely reflects the relatively small number of transcriptomes per treatment (*n* = 3–4), which renders our estimates of the number of differentially expressed genes conservative, although comparable sample sizes revealed hundreds sex- and age-biased genes. The relatively low number of testosterone-responsive genes may also reflect our decision to characterize gene expression 2 months after treatment. As a result, our transcriptomes likely characterize long-term changes in hepatic regulation induced by chronic elevation (or removal) of testosterone, perhaps failing to capture many of the important but relatively transient changes in gene expression that may occur in response to more short-term genomic (*cis*-regulatory) and non-genomic effects of testosterone. In addition to reducing our power to detect DEGs, fewer replicate transcriptomes per group should increase error in the estimation of mean sex, age, and treatment effects (i.e., log_2_ FC values) for individual genes. It is therefore reassuring to see that patterns of sex and age bias predict patterns of responsiveness to testosterone, and *vice versa*, despite the conservative nature of such tests when based on fewer biological replicates. We also found that dispersion estimates from our DEG analyses (common dispersion = ∼0.10; data not shown), which are indicative of the biological coefficient of variance, were in line with values expected from outbred populations (McCarthy, Chen and Smyth, 2012), suggesting that within-group variance among replicates did not have a disproportionate influence on our results.

Second, it is possible that some of the effects of testosterone that we observed are due to estrogenic signaling following local conversion of exogenous testosterone to estradiol. Although we cannot confirm androgenic signaling as the mechanism of responsiveness for any given gene or pathway, we have previously found that phenotypic effects of testosterone (i.e., enhanced growth and development of the dewlap) are induced by 5α-dihydrotestosterone, which cannot be aromatized to estradiol (R.M. Cox, T.N. Wittman, A. Walsh, unpublished data). As such, it is likely that many of the transcriptional effects we observed are mediated by androgenic signaling, although we cannot directly confirm this on a gene-by-gene basis.

Finally, our analyses cannot determine the relative extent to which the observed effects of testosterone on transcription in the liver are mediated by direct *cis*-regulation (i.e., genes in the liver with proximate androgen response elements, AREs) vs. *trans*-regulation either within the liver or *via* upstream signals that act on the liver (e.g., growth hormone, GH). Our previous attempts to link testosterone-responsive genes in the brown anole to putative AREs predicted from *in silico* analysis of the genome of a congener (*Anolis carolinensis*) found little evidence for direct *cis*-regulation *via* AREs (Cox, 2019). While this likely reflects the limitations of using the genome of a congener and relying on *in silico* predictions, it also suggests that many effects of testosterone on hepatic transcription that we observed may be indirect. Sex-biased gene expression in the liver is best described in rodent models and is often driven by sex-specific patterns of GH secretion from the pituitary. Male-typical GH pulses dictate sex-specific activity of transcriptional regulators in the liver, which in turn are responsible for many sex differences in transcription, growth rate, and body size (Udy et al., 1997; Davey et al., 1999; Clodfelter et al., 2006; Lau-Corona, Suvorov and Waxman, 2017). Androgens influence this process by masculinizing GH regulatory pathway structure in the hypothalamus during neonatal development, and by potentiating GH release and action from the pituitary during puberty and in adulthood (Chowen, Frago and Argente, 2004; Meinhardt and Ho, 2007). It remains to be seen whether and how direct, *cis*-regulatory effects of testosterone in the liver may contribute to patterns of sex-biased gene expression independent of upstream effects on GH secretion, and effects of testosterone on the transcriptional networks downstream of GH signaling remain poorly studied outside of rodents.

The similarity in liver transcriptomes that we observed between juvenile females and males mirrors the lower levels of phenotypic sexual dimorphism observed in juvenile brown anoles (Cox et al., 2009b; Sanger et al., 2014; Cox et al., 2015, 2017). In this species, the transition from juvenile to subadult is accompanied by an acceleration in growth of males, relative to that of females, and this sexual divergence occurs concomitantly with an increase in sex-biased gene expression in signaling pathways related to growth, metabolism, and cell proliferation (i.e., growth hormone/insulin-like growth factor (GH/IGF) and mechanistic target of rapamycin (mTOR) pathways) in the liver (Cox et al., 2017). Testosterone stimulates growth in length and mass as well as the expression of genes of these growth-regulatory pathways (Cox et al., 2015, 2017). Our finding that translation pathways were enriched for subadult-biased DEGs in males is likely also a reflection of the acceleration in male growth that underlies the emergence of sexual size dimorphism in brown anoles. Although we did not specifically detect enrichment of GH/IGF and mTOR signaling pathways with sex- or age-biased DEGs, these pathways contribute to protein synthesis and anabolism (Wang and Proud, 2006; Møller and Jørgensen, 2009; Yoshida and Delafontaine, 2020). Thus, enrichment of translation and protein synthesis pathways with genes that were upregulated with age in males, but not in females, is also consistent with the idea that developmental changes within males drive sexual divergence at both phenotypic and transcriptomic levels. Other pathways with potential roles in growth, such as ribosomal biogenesis and RNA processing, were enriched with age-biased genes in both sexes, although statistical enrichment of these pathways was more robust in males than in females.

Collectively, our results illustrate how the developmental emergence of sex-biased gene expression can be usefully explored by using the same transcriptomic data to simultaneously characterize ontogenetic changes within each sex. In the case of the brown anole liver, this approach revealed that ontogenetic changes in gene expression were much more pronounced in males than in females, similar to what has been observed in the mouse liver (Conforto and Waxman, 2012). Determining whether this is a general feature of sex-specific gene regulation or (more likely) one that varies by species, tissue, and ontogenetic stage will require comparable analyses in other systems. For the brown anole liver, however, the high degree of age-biased gene expression that we observed in males is consistent with the observation that male morphological development appears to deviate more from the juvenile pattern as maturation progresses. In our study, the direction of sexual asymmetry in age-biased gene expression also raised the hypothesis that pleiotropic regulators of gene expression that are characteristic of males, such as androgens, are important for the development of sex-biased gene expression. In direct support of this hypothesis, we found that several different experimental characterizations of transcriptomic responsiveness to testosterone each independently predicted the direction of sex-biased expression in subadults and age-biased expression in males. Nonetheless, patterns of age-biased gene expression were largely congruent in both sexes, suggesting that androgenic regulation primarily accentuates sex bias in gene expression and magnifies the degree of age bias within males, rather than reversing the direction of age bias between sexes. Future work that links patterns of sex- and age-biased expression to features of the genomic architecture that enable endocrine regulation, such as androgen and estrogen response elements, will help clarify the extent to which testosterone exerts its regulatory effects upstream of the liver (e.g., *via* GH secretion), parse *cis* vs. *trans* regulatory effects of testosterone on gene expression in the liver, and identify the genetic targets of endocrine signaling.

## Data Availability

The datasets presented in this study can be found in online repositories. The names of the repositories and accession number(s) can be found below: https://www.ncbi.nlm.nih.gov/, PRJNA348684, PRJNA833864.
