# Supplementary material for: Ontogenetic Change in Male Expression of Testosterone-Responsive Genes Contributes to the Emergence of Sex-Biased Gene Expression in Anolis sagrei
Source: Front Physiol. 2022 Jun 2;13:886973. doi: 10.3389/fphys.2022.886973 (PMC9203151; doi:10.3389/fphys.2022.886973)

Supplementary Material

Ontogenetic change in male expression of testosterone-responsive genes contributes to the emergence of sex-biased gene expression in *Anolis sagrei*

Matthew D. Hale[1], Christopher D. Robinson[1], Christian L. Cox[2], and Robert M. Cox[1]

Supplementary Files

DEG_supplement.xlsx (separate file) provides ENSACAT gene IDs, gene symbols, $\log_2$ (fold change), $\log_2$ (mean counts per million), *F* statistics, uncorrected *P* values, false discovery rate-corrected (FDR) *P* values, and $\log_{10}$ (*P*) for 13,464 individual genes (transcripts) across each of the 8 experimental contrasts used for analysis of differential gene expression. Gene symbols were obtained by converting original transcript identifiers (AnoCar2.0; Ensembl Release 75) to current (AnoCar2.0v2; Ensembl Release 106) identifiers via biomaRt. HGNC gene symbols were included where available for any transcript with a stable identifier across both releases.

Supplementary Tables

Table S1. Number of age-biased DEGs identified in each sex using all available data (n = 3 for juvenile females, n = 4 for other groups) and using four subset models in which a single juvenile male library was removed to balance sample size across sexes. "Fold" quantifies the relative magnitude of the sex difference in number of DEGs in each analysis (number of DEGs in males divided by number of DEGs in females).

Sex	Age bias	All data	Subset 1	Subset 2	Subset 3	Subset 4
Male	Juvenile-biased	977	741	799	787	969
	Subadult-biased	893	639	605	594	924
	Total age-biased	1870	1380	1404	1381	1893
Female	Juvenile-biased	127	129	116	120	150
	Subadult-biased	133	136	122	126	153
	Total age-biased	260	265	238	246	303
Fold (M/F)	Juvenile-biased	7.69	5.74	6.88	6.56	6.46
	Subadult-biased	6.71	4.69	4.96	4.71	6.04
	Total age-biased	7.19	5.21	5.90	5.61	6.24

Table S2. **Statistical tests for overlap in sex-biased gene expression in subadults, age-biased gene expression in females, and age-biased gene expression in males.** JF = juvenile female, JM = juvenile male, SF = subadult female, SM = subadult male. "Figure" indicates the corresponding visualization of statistical results. "DEGs" indicates the comparison used to generate differentially expressed genes (e.g., SF < SM in the first row indicates that genes used in the statistical comparisons are DEGs with male-biased expression in subadults). "Prediction" indicates the comparison used to derive $\log_2$ (FC) values for these DEGs and the direction of the predicted statistical result (e.g., JF > SF in the first row indicates that DEGs that are male-biased in subadults are predicted to be juvenile-biased in females – all observed results corresponded to the direction of these predictions). Results are reported for parametric tests of mean $\log_2$ (FC) values using Welch's one-sample *t*-tests, and for non-parametric tests of median $\log_2$ (FC) values using Wilcoxon signed-rank tests (null hypothesis: mean or median $\log_2$ (FC) = 0). "d.f." = degrees of freedom (number of DEGs – 1). Unadjusted *P*-values are shown. All significant ($P < 0.05$) results remained significant following Bonferroni adjustment for 12 comparisons. Asterisks next to the figure number indicate comparisons that are statistically non-independent due to a shared group between DEG and Prediction columns.

			**Welch's *t***			**Wilcoxon Signed Rank**	
**Figure**	**DEGs**	**Prediction**	***t***	**d.f.**	***P***	***W***	***P***
2D*	SF < SM	JF > SF	-9.03	265	< 0.0001	-23257	< 0.0001
	SF > SM	JF < SF	11.23	211	< 0.0001	20190	< 0.0001
2E*	JF < SF	SF > SM	-10.82	132	< 0.0001	-8261	< 0.0001
	JF > SF	SF < SM	6.32	126	< 0.0001	4602	< 0.0001
2F*	JM < SM	SF < SM	20.04	892	< 0.0001	296817	< 0.0001
	JM > SM	SF > SM	-18.52	976	< 0.0001	-313379	< 0.0001
2G*	SF < SM	JM < SM	14.84	265	< 0.0001	31357	< 0.0001
	SF > SM	JM > SM	-8.01	211	< 0.0001	-13100	< 0.0001
2H	JF < SF	JM < SM	13.19	132	< 0.0001	7921	< 0.0001
	JF > SF	JM > SM	-15.17	126	< 0.0001	-7606	< 0.0001
2I	JM < SM	JF < SF	22.82	892	< 0.0001	313913	< 0.0001
	JM > SM	JF > SF	-32.08	976	< 0.0001	-418215	< 0.0001

Table S3. Statistical tests for correspondence across different experimental contrasts for responsiveness to testosterone. JF = juvenile female, JFT = juvenile female + testosterone implant, JM = juvenile male, JMT = juvenile male + testosterone implant, SM = subadult male, SMC = castrated subadult male, SMCT = castrated subadult male + testosterone implant. "Figure" indicates the corresponding visualization of statistical results. "DEGs" indicates the comparison used to generate differentially expressed genes (see Table S2 for example). "Prediction" indicates the comparison used to derive $\log_2$ (FC) values for these DEGs and the direction of the predicted statistical result (see Table S2 for example). Results are shown for parametric tests of mean $\log_2$ (FC) values using Welch's one-sample *t*-tests and for non-parametric tests of median $\log_2$ (FC) values using Wilcoxon signed-rank tests (null hypothesis: mean or median $\log_2$ (FC) = 0). "d.f." = degrees of freedom (number of DEGs – 1). Unadjusted *P*-values are shown. *Italics* indicates results that were no longer significant following Bonferroni adjustment for 24 comparisons. Asterisks next to the figure number indicate comparisons that are statistically non-independent due to a shared group between DEG and Prediction columns.

			**Welch's *t***			**Wilcoxon Sign Rank**	
**Figure**	**DEGs**	**Prediction**	***t***	**df**	***P***	***W***	***P***
S3B	JM < JMT	JF < JFT	*1.72*	*95*	*0.0887*	*1356*	*0.0133*
	JM > JMT	JF > JFT	*-2.27*	*156*	*0.0243*	*-2665*	*0.0196*
S3C	SMC < SM	JF < JFT	5.97	200	< 0.0001	9571	< 0.0001
	SMC > SM	JF > JFT	*-1.97*	*117*	*0.0509*	*-1511*	*0.0426*
S3D	SMC < SMCT	JF < JFT	5.98	224	< 0.0001	11515	< 0.0001
	SMC > SMCT	JF > JFT	-3.23	216	0.0014	-6519	0.0004
S3E	JF < JFT	JM < JMT	*1.96*	*95*	*0.0529*	*1466*	*0.0074*
	JF > JFT	JM > JMT	-3.38	81	0.0011	-1565	0.0003
S3G	SMC < SM	JM < JMT	5.74	200	< 0.0001	9533	< 0.0001
	SMC > SM	JM > JMT	-5.04	117	< 0.0001	-3837	< 0.0001
S3H	SMC < SMCT	JM < JMT	4.78	224	< 0.0001	14441	< 0.0001
	SMC > SMCT	JM > JMT	-4.11	216	< 0.0001	-7155	0.0001
S3I	JF < JFT	SMC < SM	6.41	95	< 0.0001	3388	< 0.0001
	JF > JFT	SMC > SM	-3.68	81	0.0004	-1507	0.0004
S3J	JM < JMT	SMC < SM	5.00	95	< 0.0001	2830	< 0.0001
	JM > JMT	SMC > SM	-4.64	156	< 0.0001	-5257	< 0.0001
S3L*	SMC < SMCT	SMC < SM	15.79	224	< 0.0001	25155	< 0.0001
	SMC > SMCT	SMC > SM	-13.85	216	< 0.0001	-21081	< 0.0001
S3M	JF < JFT	SMC < SMCT	5.61	95	< 0.0001	2988	< 0.0001
	JF > JFT	SMC > SMCT	-3.85	81	0.0002	-1705	< 0.0001
S3N	JM < JMT	SMC < SMCT	4.37	95	< 0.0001	2578	< 0.0001
	JM > JMT	SMC > SMCT	-3.27	156	0.0013	-6961	< 0.0001
S3O*	SMC < SM	SMC < SMCT	12.24	200	< 0.0001	18751	< 0.0001
	SMC > SM	SMC > SMCT	-14.25	117	< 0.0001	-6899	< 0.0001

Table S4. Statistical tests of whether responsiveness to testosterone predicts sex-biased gene expression in subadults and age-biased gene expression in males. JF = juvenile female, JFT = juvenile female + testosterone implant, JM = juvenile male, JMT = juvenile male + testosterone implant, SF = subadult female, SM = subadult male, SMC = castrated subadult male, SMCT = castrated subadult male + testosterone implant. “Figure” indicates the corresponding visualization of statistical results. “DEGs” indicates the comparison used to generate differentially expressed genes (see Table S2 for example). “Prediction” indicates the comparison used to derive $\log_2$ (FC) values for these DEGs and the direction of the predicted statistical result (see Table S2 for example). Results are reported for parametric tests of mean $\log_2$ (FC) values using Welch’s one-sample *t*-tests, and for non-parametric tests of median $\log_2$ (FC) values using Wilcoxon signed-rank tests (null hypothesis: mean or median $\log_2$ (FC) = 0). “d.f.” = degrees of freedom (number of DEGs – 1). Unadjusted *P*-values are shown. All significant ($P < 0.05$) results remained significant following Bonferroni adjustment for 16 comparisons. Asterisks next to the figure number indicate comparisons that are statistically non-independent due to a shared group between DEG and Prediction columns.

			**Welch's *t***			**Wilcoxon Signed Rank**	
**Figure**	**DEGs**	**Prediction**	***t***	**df**	***P***	***W***	***P***
4E	JF < JFT	SF > SM	7.41	95	< 0.0001	3814	< 0.0001
	JF > JFT	SF < SM	-4.15	81	< 0.0001	-2027	< 0.0001
4F	JM < JMT	SF > SM	5.02	95	< 0.0001	2586	< 0.0001
	JM > JMT	SF < SM	-0.01	156	0.9895	189	0.8691
4G*	SMC < SM	SF > SM	15.68	200	< 0.0001	19785	< 0.0001
	SMC > SM	SF < SM	-10.22	117	< 0.0001	-5967	< 0.0001
4H	SMC < SMCT	SF > SM	6.59	224	< 0.0001	12415	< 0.0001
	SMC > SMCT	SF < SM	1.45	216	0.1492	1707	0.3569
4I	JF < JFT	JM < SM	5.53	95	< 0.0001	2840	< 0.0001
	JF > JFT	JM > SM	-3.49	81	0.0008	-1633	0.0002
4J*	JM < JMT	JM < SM	10.22	95	< 0.0001	4120	< 0.0001
	JM > JMT	JM > SM	-13.84	156	< 0.0001	-11337	< 0.0001
4K*	SMC < SM	JM < SM	15.32	200	< 0.0001	19523	< 0.0001
	SMC > SM	JM > SM	-12.15	117	< 0.0001	-6431	< 0.0001
4L	SMC < SMCT	JM < SM	7.95	224	< 0.0001	17279	< 0.0001
	SMC > SMCT	JM > SM	-5.76	216	< 0.0001	-10665	< 0.0001

Table S5. Statistical tests of whether sex-biased gene expression in subadults and age-biased gene expression in males predict responsiveness to testosterone. JF = juvenile female, JFT = juvenile female + testosterone implant, JM = juvenile male, JMT = juvenile male + testosterone implant, SF = subadult female, SM = subadult male, SMC = castrated subadult male, SMCT = castrated subadult male + testosterone implant. "Figure" indicates the corresponding visualization of statistical results. "DEGs" indicates the comparison used to generate differentially expressed genes (see Table S2 for example). "Prediction" indicates the comparison used to derive $\log_2$ (FC) values for these DEGs and the direction of the predicted statistical result (see Table S2 for example). Results are reported for parametric tests of mean $\log_2$ (FC) values using Welch's one-sample *t*-tests and for non-parametric tests of median $\log_2$ (FC) values using Wilcoxon signed-rank tests (null hypothesis: mean or median $\log_2$ (FC) = 0). "d.f." = degrees of freedom (number of DEGs – 1). Unadjusted *P*-values are shown. *Italics* indicates results that were no longer significant following Bonferroni adjustment for 16 comparisons. Asterisks next to the figure number indicate comparisons that are statistically non-independent due to a shared group between DEG and Prediction columns.

			**Welch's *t***			**Wilcoxon Signed Rank**	
**Figure**	**DEGs**	**Prediction**	***t***	**df**	***P***	***W***	***P***
S4A	JM < SM	JF < JFT	*2.40*	*892*	*0.0166*	-9011	0.5590
	JM > SM	JF > JFT	-3.18	976	0.0015	-61985	0.0004
S4B*	JM < SM	JM < JMT	16.44	892	< 0.0001	260003	< 0.0001
	JM > SM	JM > JMT	-18.33	976	< 0.0001	-343453	< 0.0001
S4C*	JM < SM	SMC < SM	21.59	892	< 0.0001	358523	< 0.0001
	JM > SM	SMC > SM	-22.16	976	< 0.0001	-379081	< 0.0001
S4D	JM < SM	SMC < SMCT	15.03	892	< 0.0001	249243	< 0.0001
	JM > SM	SMC > SMCT	-12.99	976	< 0.0001	-271145	< 0.0001
S4E	SF < SM	JF < JFT	8.26	265	< 0.0001	20691	< 0.0001
	SF > SM	JF > JFT	-3.17	211	0.0018	-5400	0.0025
S4F	SF < SM	JM < JMT	2.81	265	< 0.0001	8375	< 0.0001
	SF > SM	JM > JMT	-1.30	211	0.1939	928	0.6042
S4G*	SF < SM	SMC < SM	13.60	265	< 0.0001	32763	< 0.0001
	SF > SM	SMC > SM	-6.69	211	< 0.0001	-12914	< 0.0001
S4H	SF < SM	SMC < SMCT	3.15	265	0.0018	9477	0.0002
	SF > SM	SMC > SMCT	-0.66	211	0.5107	26	0.9888

Supplementary Figures

Figure S1. Gene ontology (GO) analysis of sex-biased and age-biased genes. GO terms for functional pathways (gene ontology: biological function) enriched for (**A**) male-biased DEGs in comparisons of subadult females and males (no pathways were significantly enriched for female-biased DEGs), and (**B**) age-biased genes in comparisons of juvenile and subadult males or juvenile and subadult females. Blue bars correspond to pathways enriched for subadult-biased genes in males, turquoise bars correspond to pathways enriched for juvenile-biased genes in males, and red bars correspond to pathways enriched for subadult-biased genes in females. The x-axis reports the -log(10) transformation of the g:SCS-adjusted p-value of pathway enrichment.

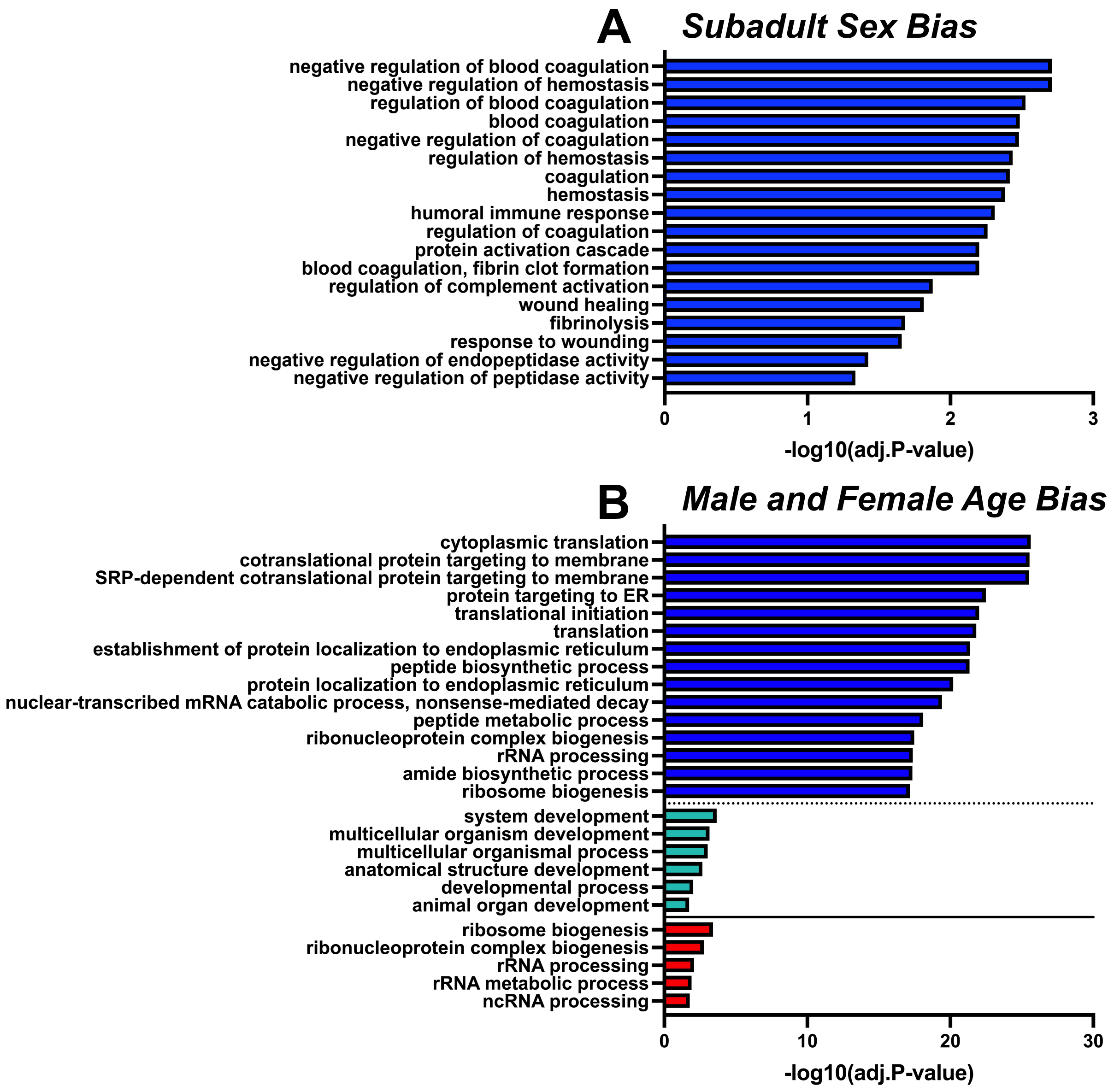

Figure S2. Overlap in age- and sex-biased genes and testosterone-responsive genes inferred to be differentially expressed. (A) Three-way Venn diagram depicting shared and unique DEGs across age sex bias in subadults, age bias in females, and age bias in males. **(B)** Four-way Venn diagram depicting shared and unique DEGs across four experimental comparisons used to identify testosterone-responsive genes.

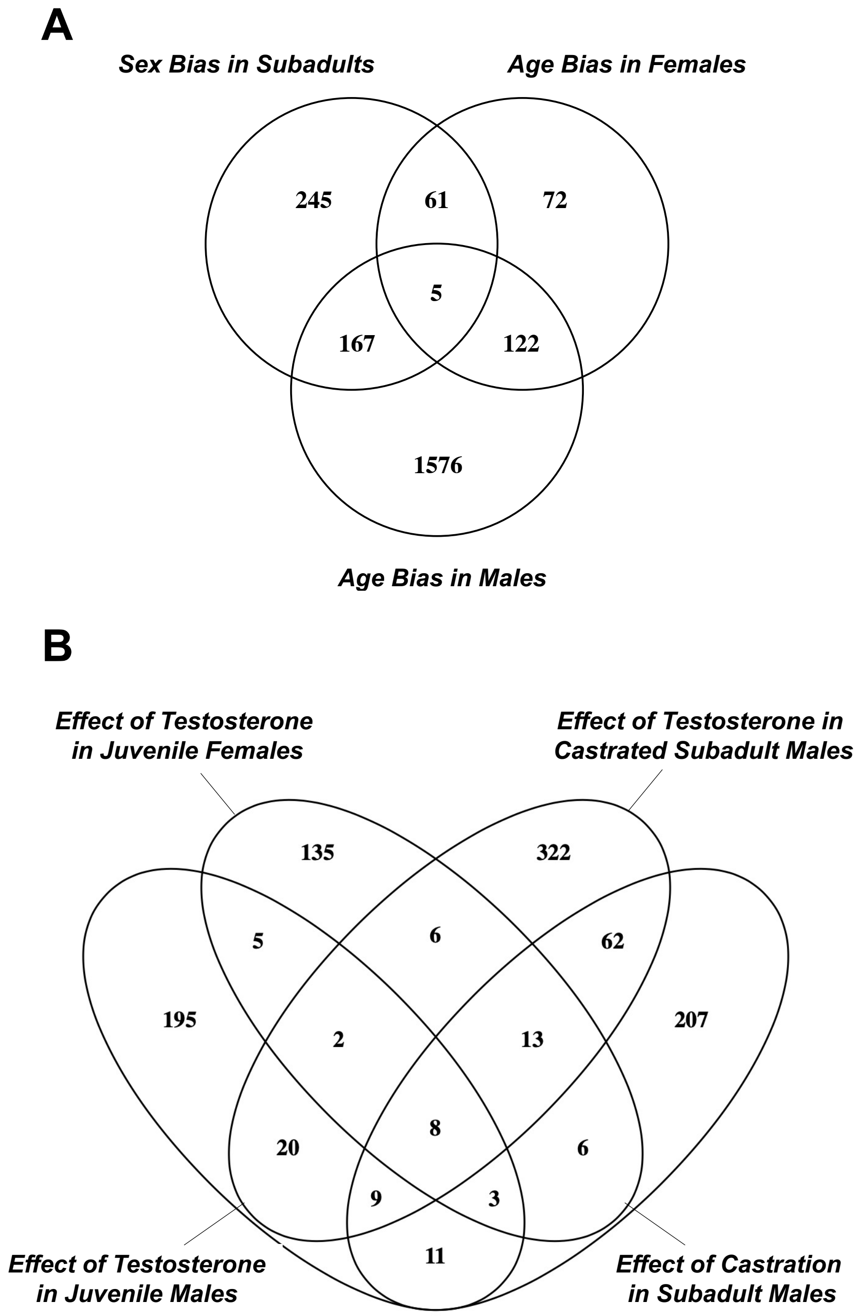

Figure S3. Overlap in testosterone-responsive genes identified by four experimental contrasts. Volcano plots on the diagonal illustrate differentially expressed genes (DEGs, $P < 0.01$) based on comparisons of (**A**) juvenile females with empty versus testosterone implants (JF vs JFT), (**F**) juvenile males with empty versus testosterone implants (JM vs JMT), (**K**) castrated versus intact subadult males (SMC vs SM), and (**P**) castrated subadult males with empty versus testosterone implants (SMC vs SMCT). In the off-diagonal panels, DEGs identified by the contrast on the diagonal in that column are mapped onto volcano plots describing effects of testosterone for other experimental contrasts, as indicated by labels to the right of each row. Volcano plots are the same within each row and DEGs are the same within each column. Box-and-whisker plots above each panel report the median (line), upper 75% and lower 25% (box), and upper 95% and lower 5% (whiskers) for the $\log_2$ (fold change) values of each category of DEG when mapped onto the corresponding plot. Asterisks indicate mean $\log_2$ (FC) values significantly different from zero following Bonferroni correction for 24 comparisons (adjusted $P < 0.0021$). See Table S3 for corresponding statistical results. Red asterisks indicate tests for which the classification of DEGs is not statistically independent of the $\log_2$ (FC) values on the volcano plot because the same group is included in each comparison.

Fig. S3 (legend on preceding page)

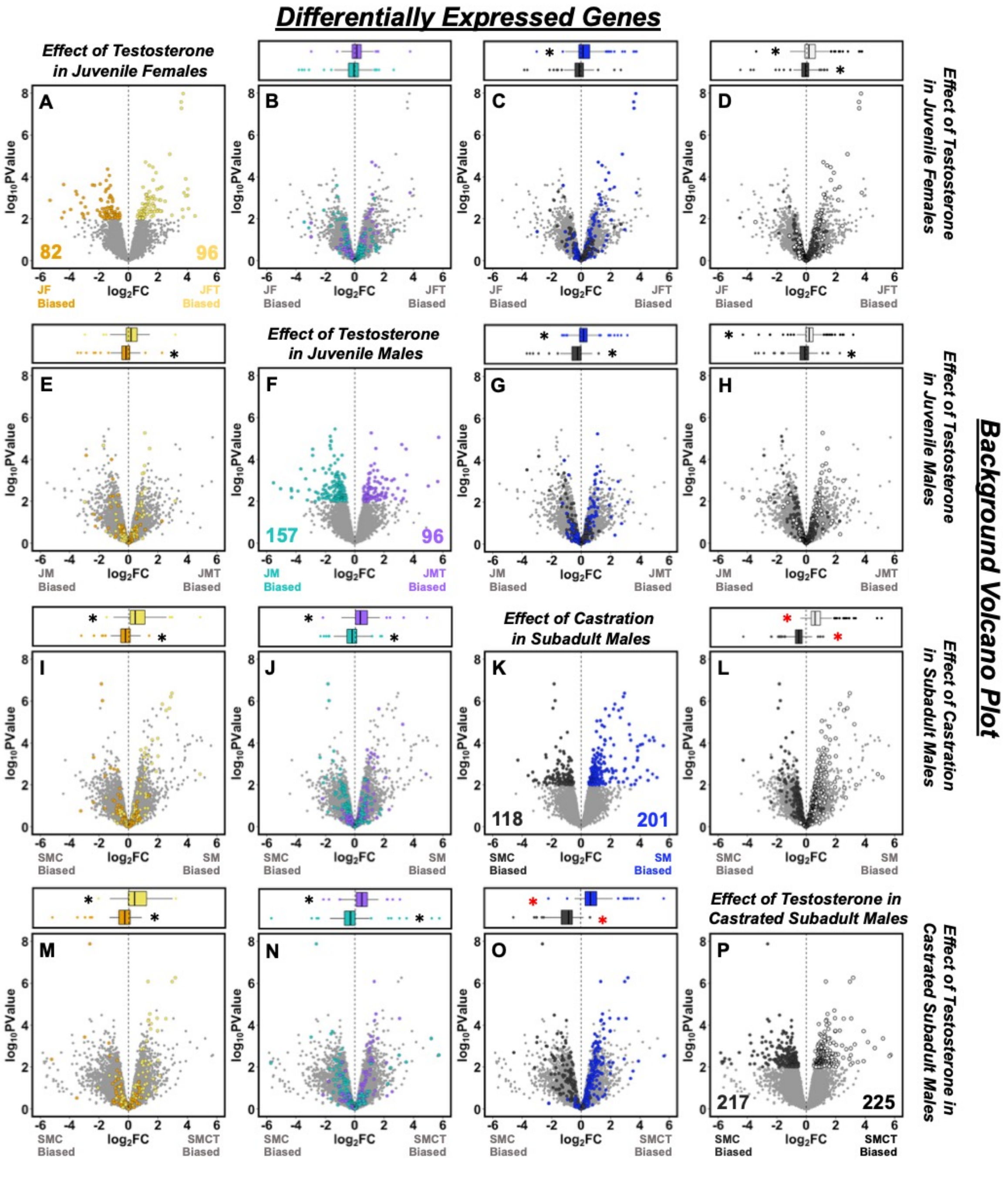

Figure S4. Overlap between sex-biased or age-biased genes and patterns of responsiveness to testosterone. Volcano plots illustrate the effects of testosterone on gene expression based on comparisons of (**A, E**) juvenile females with empty versus testosterone implants (JF vs JFT), (**B, F**) juvenile males with empty versus testosterone implants (JM vs JMT), (**C, G**) castrated versus intact subadult males (SMC vs SM), and (**D, H**) castrated subadult males with empty versus testosterone implants (SMC vs SMCT). In the top row (**A-D**), age-biased genes in males (DEGs from **Fig. 1E**, FDR < 0.05) are plotted onto each of the volcano plots describing responsiveness to testosterone, with DEGs in color and other genes in gray. In the bottom row (**E-H**), sex-biased genes in subadults (DEGs from **Fig. 1B**, FDR < 0.05) are plotted onto each of the same volcano plots. Box-and-whisker plots above each panel report the median (line), upper 75% and lower 25% (box), and upper 95% and lower 5% (whiskers) for the $\log_2$ (fold change) values of each category of DEG when mapped onto the corresponding volcano plot. Asterisks indicate mean $\log_2$ (FC) values significantly different from zero following Bonferroni correction for 16 comparisons (adjusted $P < 0.0031$). See Table S5 for statistical results. Red asterisks indicate tests for which the classification of DEGs is not statistically independent of the $\log_2$ (FC) values on the volcano plot because the same group is included in each comparison.

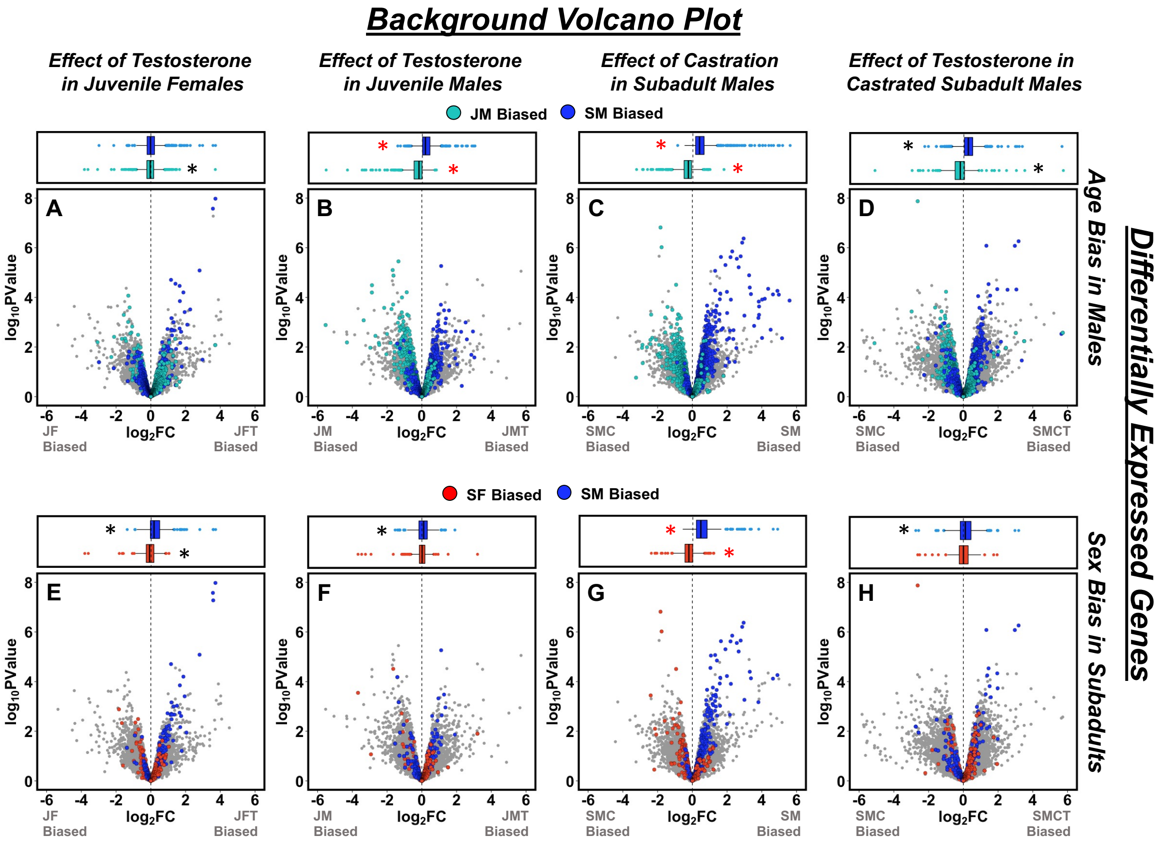

Supplement: Supplementary file 1 [file DataSheet1.pdf]
